# Optimizing deep brain stimulation based on isostable amplitude in essential tremor patient models

**DOI:** 10.1088/1741-2552/abd90d

**Published:** 2021-04-06

**Authors:** Benoit Duchet, Gihan Weerasinghe, Christian Bick, Rafal Bogacz

**Affiliations:** 1 Nuffield Department of Clinical Neurosciences, University of Oxford, Oxford, United Kingdom; 2 MRC Brain Network Dynamics Unit, University of Oxford, Oxford, United Kingdom; 3 Oxford Centre for Industrial and Applied Mathematics, Mathematical Institute, University of Oxford, Oxford, United Kingdom; 4 Centre for Systems, Dynamics, and Control and Department of Mathematics, University of Exeter, Exeter, United Kingdom; 5 EPSRC Centre for Predictive Modelling in Healthcare, University of Exeter, Exeter, United Kingdom

**Keywords:** deep brain stimulation, essential tremor, isostable amplitude, Hilbert amplitude, stimulation strategy, pathological oscillations

## Abstract

**Objective:**

Deep brain stimulation is a treatment for medically refractory essential tremor. To improve the therapy, closed-loop approaches are designed to deliver stimulation according to the system’s state, which is constantly monitored by recording a pathological signal associated with symptoms (e.g. brain signal or limb tremor). Since the space of possible closed-loop stimulation strategies is vast and cannot be fully explored experimentally, how to stimulate according to the state should be informed by modeling. A typical modeling goal is to design a stimulation strategy that aims to maximally reduce the Hilbert amplitude of the pathological signal in order to minimize symptoms. Isostables provide a notion of amplitude related to convergence time to the attractor, which can be beneficial in model-based control problems. However, how isostable and Hilbert amplitudes compare when optimizing the amplitude response to stimulation in models constrained by data is unknown.

**Approach:**

We formulate a simple closed-loop stimulation strategy based on models previously fitted to phase-locked deep brain stimulation data from essential tremor patients. We compare the performance of this strategy in suppressing oscillatory power when based on Hilbert amplitude and when based on isostable amplitude. We also compare performance to phase-locked stimulation and open-loop high-frequency stimulation.

**Main results:**

For our closed-loop phase space stimulation strategy, stimulation based on isostable amplitude is significantly more effective than stimulation based on Hilbert amplitude when amplitude field computation time is limited to minutes. Performance is similar when there are no constraints, however constraints on computation time are expected in clinical applications. Even when computation time is limited to minutes, closed-loop phase space stimulation based on isostable amplitude is advantageous compared to phase-locked stimulation, and is more efficient than high-frequency stimulation.

**Significance:**

Our results suggest a potential benefit to using isostable amplitude more broadly for model-based optimization of stimulation in neurological disorders.

## 1 Introduction

Essential tremor (ET) is a neurological disorder believed to originate from aberrant neural synchrony in the cerebellar-thalamic-cortical pathway [1]. ET is characterized by a tremor of the upper limbs, and tremulous hand movements are coherent with thalamic activity [2–4]. When medications are ineffective or not tolerated, high-frequency deep brain stimulation (DBS) of the thalamus is an effective therapy [5–7]. In its clinically available version, DBS delivers pulses of electrical stimulation at about 130 Hz via electrodes implemented deep into the brain. Reported side effects of high-frequency (HF) thalamic DBS include speech impairment, gait disorders, and abnormal dermal sensations [8].

Such side-effects due to HF stimulation, the inability to adapt stimulation to symptom severity, frequent battery replacement surgeries, as well as habituation in some patients (diminishing stimulation efficacy [9]) are some of the key limitations of HF DBS in ET. New closed-loop DBS strategies have therefore been explored to stimulate less while preserving or even improving clinical efficacy. Examples of closed-loop strategies include phase-locked DBS, where stimulation is delivered according to the phase of pathological oscillations [10, 11], and adaptive DBS, where stimulation is triggered based on the amplitude of pathological oscillations [12–17]. There is no consensus on how to stimulate to best minimize aberrant synchrony, and experimental testing is limited because patients fatigue quickly.

Among model-based control approaches in neurological disorders and in particular in movement disorders, strategies based on the phase dependence of the response to stimulation, strategies based on optimal control, and strategies based on feedback control have been particularly influential. Phase dependence of the response to stimulation has been utilized both at the individual oscillator level and at the population level. The knowledge of individual oscillator phase response is used in [18–20] to design stimuli aiming at desynchronizing coupled oscillators. At the neural population level, a strategy consisting in providing bursts of stimulation phase-locked to the phase of population activity was suggested in [21]. Recently, a Bayesian adaptive dual control algorithm was designed to learn how to best stimulate according to the phase and power of recorded oscillatory activity [22]. Optimal control has been used to steer neural systems to a particular target set, such as the phaseless set of a coupled oscillator model [23], or a target set determined through bifurcation analysis of a neuron model [24]. Some feedback control strategies in neurological diseases have relied on the online estimation of a model [14, 25, 26]. Model predictive control has been applied to Parkinson’s disease (PD) using an extended autoregressive model [27], and various forms of delayed feedback have also been investigated [28, 29]. Further review can be found for instance in [30,31].

Modeling studies such as [20, 32] have been carried out to provide insights on how to effectively reduce the Hilbert amplitude of the pathological signal with stimulation in ET. The Hilbert amplitude is one of the most commonly used measures of instantaneous amplitude in neurophysiological data [10, 11, 33–36]. It is defined as the modulus of the analytic signal, and gives the amplitude of the envelope of narrow-band signals.

On the other hand, the notion of isostables, which define an amplitude coordinate, has been applied with great success to control the state of various systems. Trajectories starting on the same isostable approach the attractor synchronously (isostables define a set of coordinates with codimension one associated with the decaying eigenvalues of an attractor [37]). Isostables have been defined both for limit cycle dynamics [38, 39] and fixed point (FP) dynamics [37], and have been applied to control problems in both cases. Outside of neuroscience, examples include stopping cardiac alternans [40], controlling non-linear flows [41], and optimal convergence to a stable FP [42]. In [43], a stimulation strategy is designed based on isostable reduction to desynchronize coupled neurons modeling pathological synchronization in PD. Besides isostables, methods to define control strategies based on the offline analysis of phase space have been suggested for instance in [44, 45].

How isostable and Hilbert amplitudes compare when optimizing the amplitude response to stimulation in models constrained by data is unknown. Since isostable amplitude delineates convergence time to the attractor, it provides information on the longterm behavior of the system, which may by useful in model-based studies of DBS. In this work, using models fitted in [32] to phase-locked stimulation data from ET patients [10], we ask the question ‘Can phase space stimulation based on isostable amplitude be advantageous compared to phase space stimulation based on Hilbert amplitude, and compared to simpler stimulation strategies?’ Because of the transient character of tremor, the deterministic part of best model fits from [32] gave rise to stable foci. We will therefore consider isostable amplitude of stable foci [37].

On the basis of stimulation strategies benefiting from the knowledge of phase space amplitude fields, we show that stimulation based on isostable amplitude can be beneficial compared to stimulation based on Hilbert amplitude when computation time is limited to minutes. We also compare the performance of stimulation based on isostable amplitude to phase-locked stimulation and HF stimulation. Our results open opportunities for model-based optimization of stimulation in neurological disorders where stimulation aims at minimizing the amplitude of an output.

## 2 Methods

To compare phase space stimulation strategies, we consider previously fitted models, and their isostable and Hilbert amplitude fields. We also implement phase-locked stimulation and HF stimulation as references.

### 2.1 Wilson-Cowan models fitted to ET patient data

**Figure 1:**
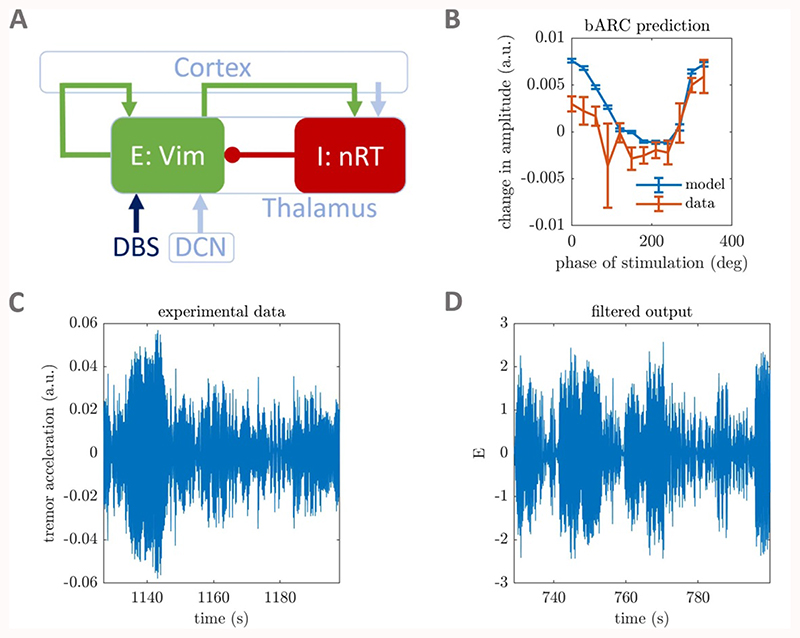
Fitting WC models to phase-locked DBS tremor data from ET patients. The WC model can describe populations thought to be involved in the generation of ET (A). Arrows denote excitatory connections or inputs, whereas circles denote inhibitory connections. The VIM is the target of DBS and also receives an input from the deep cerebellar nuclei (DCN). The self-excitatory loop of the VIM, as well as the excitatory connection from VIM to nRT are via cortex. Data and fitted model bARCs for patient 5 are shown in panel (B) (patient numbers referto [10]). Filtered tremor acceleration data (C) and fitted model output (D) are also shown for patient 5. All four panels have been adapted from [32] under the creative_commons_CC_BY_4.0_licence.

We rely on neural mass models previously fitted in [32] to tremor data from ET patients receiving phase- locked DBS [10]. In the latter study, a brief burst of stimulation was delivered to the thalamus once per tremor oscillation cycle, locked to the phase of tremor recorded from an accelerometer fitted to each patient (the data are available online [46]). In this section, we review the results of [32] relevant to the present study. The neural masses considered are twodimensional non-linear Wilson–Cowan (WC) models [47] with Gaussian white noise. The WC model depicts the interactions of a population of excitatory neurons, whose activity is denoted by *E,* and a population of inhibitory neurons, whose activity is denoted by *I.* The model can be mapped onto structures believed to be implicated in the generation of ET as shown in figure 1, panel (A). The ventral intermediate nucleus (VIM) of the thalamus is represented by the excitatory population, while the reticular nucleus (nRT) is represented by the inhibitory population. Their activities are given by the stochastic differential equations

(1) $$\left\{\begin{array}{l} \text{d}E=\frac{1}{\nu }(-E+f(\eta_{E}+w_{EB}E-w_{IE}I))\text{d}t+\zeta \text{d}W_{E},\\ \;\text{d}I=\frac{1}{\nu }(-I+f(\eta_{I}+w_{EI}E))\text{d}t+\zeta \text{d}W_{I}, \end{array}\right\}$$

with *w_PR_* the weight of the projection from population *‘P’* to population ‘*R*’, η_*P*_ the constant input to population ‘*P*’, and *ν* a time constant (assumed to be the same for both populations). We denote by *W_E_* and *W_I_* Wiener processes, and *ζ* is the noise standard deviation. As in [48], the function *f* is the sigmoid function

$$f(x)=\frac{1}{1+e^{-\beta (x-1)}},$$

parametrized by a steepness parameter *β*. In our (*E*, *I*) model, *E* is the key variable for two reasons. First, DBS for ET is most commonly delivered to the VIM, and we model stimulation by an increase *δ* *E* in the activity of the corresponding population (*E*). Second, since the coherence between ventral thalamic activity and wrist flexor electromyographic recordings is high [2–4], we use *E* to model the tremor signal.

We consider in the present study the best fits to the three ET patient datasets shown to have statistically significant phase and amplitude response curves in [32], namely patient 1, patient 5, and patient 6. Other patients were not fitted to in our previous study as their phase-dependent response to stimulation was not statistically significant and was considered to be dominated by noise. The fitting procedure in our previous study subjected models to the same phase-locked stimulation paradigm used in experimental data (see [32] for more details). The fits were based on features representing tremor dynamics (example of data and model tremor signals shown in panels (C) and (D) of figure 1), and the block phase response curve (bPRC, defined in [32]). In addition to other model parameters, the stimulation magnitude was fitted to best reproduce these features. Here, we denote fitted stimulation magnitudes by *δ* *E*_0_ and provide the fitted stimulation magnitude values from our previous study in table A in the supplementary material (available online at https://stacks.iop.org/JNE/18/046023/mmedia). As reported in [32], the fitted models are able to reproduce the data block amplitude response curves (bARCs, defined in [32], example in figure 1, panel (B)), without having been fit to the amplitude responses, showing that the phase-dependence of the system response to stimulation has been captured by the models. As mentioned earlier, the deterministic part of the three fitted models give rise to stable foci.

### 2.2 Isostable amplitude of stable foci in 2D

**Figure 2:**
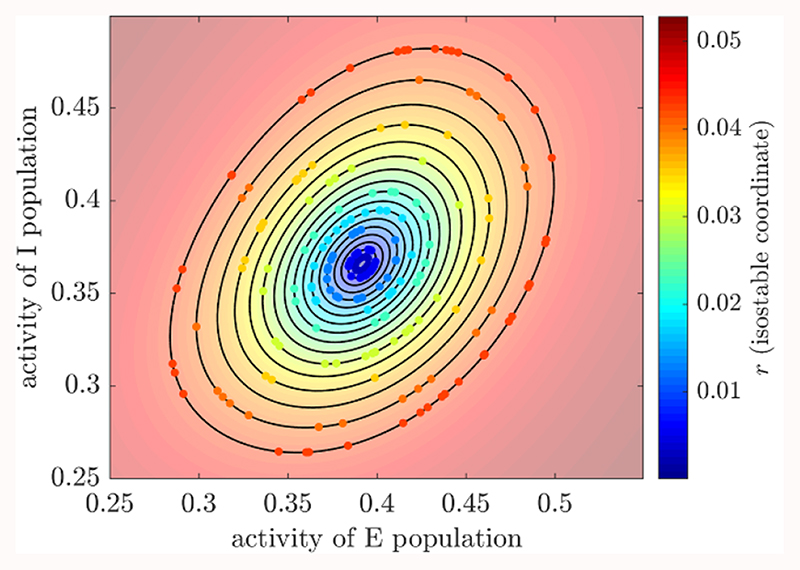
Isostables as sets of points with the same asymptotic convergence. The animation corresponding to this figure is available in the online version of this article. Trajectories starting on the same isostable are represented by dots of the same color and cross subsequent isostables at the same time. Isostables are depicted by black contours. This example shows the WC fit to patient 1. The state **X** corresponds to *(E, I).*

To introduce isostable amplitude, we summarize the concept of isostables for stable FPs presented in [37]. On an intuitive level, the isostables of a stable FP can be defined as sets of points with the same asymptotic convergence to the FP, i.e. sets of points approaching the FP synchronously [37]. This is illustrated in the animation provided in the online version of figure 2, where trajectories starting on the same isostable reach subsequent isostables at the same time. In what follows, we denote vectors in bold to distinguish scalars and vectors more easily. To provide a precise definition of isostables, let us consider a dynamical system

(2) $$\dot{\text{X}}=F(\text{X}),$$

where **X** ∈ ℝ^2^ for our purposes (see [37] for definitions in ℝ^*n*^), and the vector field *F* is analytic. The flow induced by equation (2) is denoted Φ_*t*_(**X**). We assume that *F* has a stable FP which we denote X*, with a basin of attraction B(X*). We further assume that the Jacobian *J* of *F* at X* has complex conjugate eigenvalues λ_±_ = *σ* ± *iω* with *σ* <0. We denote by **v**_1_ = **a** – *i* **b** and **v**_2_ the right normalized eigenvectors associated with λ_+_ and λ_–_, respectively. The isostable *I*_τ_ is defined in [37] when the leading eigenvalue is not real as the one-dimensional manifold

(3) $${ℐ}_{\tau }=\{\text{X}\in ℬ({\text{X}}^{*})|\exists \theta \in [0,2\pi )\;\text{s}.\text{t}.\;\underset{t\to +\infty }{\lim}e^{-\sigma t}\|\Phi_{t}(X)-{\text{X}}^{*}-ℜ[{\text{v}}_{1}e^{i(\omega t+\theta )}]e^{\sigma (t+\tau )}\|=0\},$$

where ℜ(*z*) is the real part of *z*. In equation (3), Φ_*t*_(**X**) — X* is the vector from the FP to the end point at time *t* of a trajectory with initial condition **X**. The vector ℜ[**v**_1_ *e* ^*i*^ ^(ω*t*+θ)^ *e* ^σ(*t*+τ)^ represents the asymptotic behavior at time *t* shared by trajectories with initial conditions on the isostable *I*_τ_. These vectors both converge to 0. The distance in equation (3) is therefore scaled by the increasing function of time *e* ^–σ*t*^ to make the limit meaningful. For **X** ∈ *I*_τ_, the isostable coordinate of **X** is given by *r*(**X**) = *e* ^στ^ [37], and we call *r*(**X**) isostable amplitude. To provide further intuition, *r*(**X**) is twice the modulus of the first coordinate of **Z** in the 𝕔^2^ basis (**v** ^1^, **v**_2_), where **Z** is the initial condition of a trajectory sharing the same asymptotic evolution as Φ_*t*_(**x**), but evolving according to the linearized dynamics **Ż** = *J* **Z**.

We obtain isostable amplitude in this work using a simple and efficient computation method available for FPs when the eigenvalue corresponding to the slowest direction is not real. This method is presented in Proposition 2 (ii) in [37], and we lay out how it is applied here. Isostable coordinates are computed using

(4) $$r(\text{X})\approx \frac{e^{-\sigma nT}\sqrt{{\left[\left(g_{1}o\Phi_{nT})(\text{X}\right)\right]}^{2}+{\left[\left(g_{2}o\Phi_{nT})(\text{X}\right)\right]}^{2}}}{\left|\langle \nabla g_{1}({\text{X}}^{*}),\text{a}\rangle \right|},$$

where *n* ∈ ℕ is chosen such that *n* ≫ 1 while avoiding numerical instability, ❬·, ·❭ is the standard complex inner product,$T=\frac{2\pi }{\omega }$, and *g*_1_ : ℝ^2^ ↦ ℝ and *g*_2_ : ℝ^2^ ↦ ℝ are functions called observables. We define these observables as

$$g_{1}=\langle X-X^{*},[\begin{array}{c} b_{2}\\ -b_{1} \end{array}]\rangle ,\;g_{2}=\langle \text{X}-X^{*},[\begin{array}{c} a_{2}\\ -a_{1} \end{array}]\rangle ,$$

where $a=[\begin{array}{l} a_{1}\\ a_{2} \end{array}]$ and $b=[\begin{array}{l} b_{1}\\ b_{2} \end{array}]$. These definitions satisfy the three requirements on *g*_1_ and *g*_2_ of Proposition 2 (ii) in [37]. Similarly to Laplace averages, this method permits us to compute isostable coordinates in the entire basin of attraction of the FP, but is less prone to numerical instability.

### 2.3 Obtaining Hilbert amplitude fields

Because the control goal is to reduce the Hilbert amplitude of the tremor, we use stimulation based on Hilbert amplitude fields as a basis to compare the performance of stimulation based on isostable amplitude fields. Hilbert amplitude fields can directly predict the instantaneous impact of a given stimulation pulse in phase space on the Hilbert amplitude of the tremor. We obtain Hilbert amplitude fields through averaging of numerical simulations (integration time step is 1 ms). Specifically, we randomly draw 2000 initial positions in the phase space region of interest. From these initial positions, we simulate the stochastic model (with the baseline noise level fitted to data) for 1000 periods. From each trajectory, we obtain the Hilbert amplitude of the *E* component as *|*Ẽ*(*t*) + *iH*(*Ẽ*(*t*)~)*|, where *Ẽ* is the centered *E* component, and *H* denotes the Hilbert transform. We clip 0.5% of the trajectories and the Hilbert amplitude time series at both ends to remove edge effects. To reduce the effect of noise, we lightly smooth the trajectories and Hilbert amplitude time series (moving average spanning four integration time steps). The (*E*, *I*) region of interest is discretized into 0.001 × 0.001 unit bins (0.001 × 0.0002 units for patient 5 whose *I* range is notably narrower). For each point in each trajectory, the corresponding space bin is found, and the associated Hilbert amplitude value is added to the bin. The Hilbert amplitude field over the region of interest is finally obtained by averaging Hilbert amplitude values within space bins.

To investigate differences between isostable and Hilbert amplitude based stimulation, we use two ways of centering the *E* component to obtain ^Ẽ^, resulting in two sets of Hilbert amplitude fields. The first way is to center *E* by removing its mean, which is what is commonly done to obtain Hilbert amplitude. This results in what we call a ‘mean-centered Hilbert amplitude field’. The second way is to center *E* by using information from phase space and removing E* (the first coordinate of the FP). This results in what we call a ‘FP-centered Hilbert amplitude field’.

### 2.4 Phase-locked stimulation and high-frequency stimulation

The additional information used in stimulation strategies based on amplitude fields (introduced in section 3.3) is expected to translate into increased performance compared to simpler stimulation strategies such as phase-locked stimulation. Thus we implement phase-locked stimulation as a comparison for the phase space stimulation strategy presented in section 3.3. The zero-crossing phase was used in [10] to investigate phase-locked stimulation in ET patients. We therefore provide phase-locked stimulation during model integration according to the zerocrossing phase which we estimate as in [32] (see appendix 7 therein). Briefly, we set the zero-crossing phase to zero when positive zero-crossings are detected. Between zero-crossings, we evolve the phase linearly based on a frequency determined as the inverse of the duration of the previous period. Given a selected target phase for stimulation, one pulse of stimulation is delivered as soon as the target phase is reached. In case the target phase has not yet been reached and the next positive zero-crossing is detected, it is assumed that the phase has been underestimated and stimulation is provided right then. To maximize the benefits of phase-locked stimulation, the best target phase for phase-locked stimulation is estimated from simulations consisting of 5000 s of stimulation at one of twelve phase bins (same number of bins as in [10]). The phase bin resulting in the largest decrease in the power of the pathological oscillations is taken as the target phase. This process is repeated for each patient model and each stimulation magnitude studied in section 3.4. For each condition, the stimulation magnitude is matched to what is used in phase space stimulation.

We also compare the energy delivered by HF stimulation and by our amplitude-based phase space strategies for the same clinical effect. HF stimulation is implemented by providing stimulation pulses at 130 Hz in an open-loop fashion. Using optimization (specifically the generalized pattern search algorithm [49, 50]), we determine the stimulation pulse magnitude required by HF stimulation to attain the efficacy of the best performing phase space stimulation strategy (within ±1%). This procedure is performed for each case presented in section 3.4, and six trials of 5000 s of HF stimulation are generated at each optimization step.

While HF DBS has been previously investigated in deterministic WC models in the limit cycle regime [51, 52], its mechanism of action in our stable fixed- point stochastic WC model is different. Stimulation at 130 Hz is fast enough compared to intrinsic dynamics that HF stimulation is similar in the model to stimulation provided continuously (with an appropriately reduced stimulation magnitude to maintain the total energy delivered). Thus the effect of HF stimulation can be illustrated by increasing the rate of change of the *E* population activity (originally given by equation (1)) by a constant. This modifies the vector field, and in our patient models, the vector field with HF stimulation results in trajectories

## 3 Results

We suggest a closed-loop stimulation strategy relying on phase space amplitude fields and compare the performance of this strategy when the amplitude field used portrays isostable amplitude to when it depicts Hilbert amplitude. In both cases, the goal of the stimulation strategy is to reduce as much as possible the power of pathological oscillations.

### 3.1 Full estimates of amplitude fields

**Figure 3:**
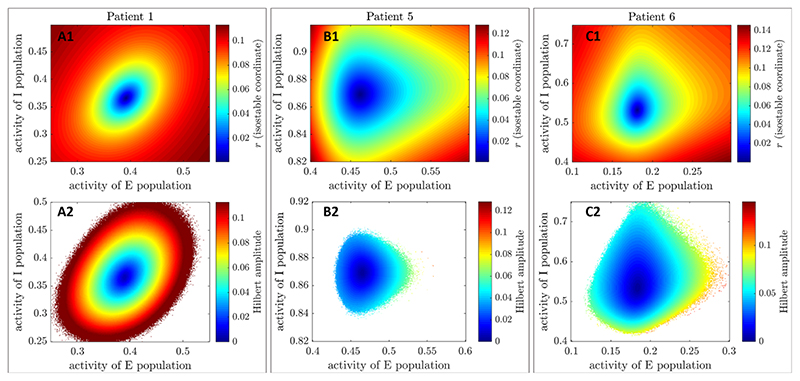
Isostable and mean-centered Hilbert amplitude fields (full estimates) for the three WC patient fits. Each column corresponds to a patient fit. Isostable amplitude fields are presented in the top row, and mean-centered Hilbert amplitude fields in the bottom row. Colour scales are matched on a patient basis. For the WC model, the state **X** corresponds to (*E*, *I*), where *E* is the activity of the excitatory population, and *I* the activity of the inhibitory population.of lower *E* amplitude when stimulation is strong enough.

Reliable estimates of isostable amplitude fields for the three WC patient fits (figure 3, panels (A1), (B1), and (C1)) were obtained using equation (4) and an explicit Runge-Kutta (4,5) method to integrate trajectories. Isostable amplitude fields are given for the deterministic version of the fitted models (i.e. with ζ = 0). As *n* is increased, convergence of isostable coordinates happens before numerical instability sets in (this was not the case with the first order Euler’s method). We used *n* = 80 for patient 1, *n* = 60 for patient 5, and *n* = 120 for patient 6. For these values, *r* has converged and there is no trace of numerical instability. Since the fitted models reproduce the particular tremor dynamics and phase-dependent response to phase-locked DBS of each individual patient [32], differences in amplitude fields between subjects should reflect patient specificities.

Hilbert amplitude fields are estimated as described in section 2.3 for the three WC patient fits. Mean-centered Hilbert amplitude fields (figure 3, panels (A2), (B2), and (C2)) are strikingly similar in shape to isostable amplitude fields. While isostable amplitude can easily be computed anywhere in the basin of attraction of the FP, Hilbert amplitude could not be determined in white zones in panels (A2), (B2) and (C2) of figure 3. FP-centered Hilbert amplitude fields (figure A in the supplementary material) are very similar to mean-centered Hilbert amplitude fields. We call the computationally intensive, high quality estimates of isostable and Hilbert amplitude fields presented in this section ‘full estimates’.

### 3.2 Quick estimates of amplitude fields

The high quality estimates of amplitude fields obtained in the previous section require up to ten hours of computation time for Hilbert amplitude fields, and on the order of one hour of computation time for isostable amplitude fields (using no more than four threads in both cases). It is of practical interest to obtain quicker estimates of amplitude fields, and to compare the performance of isostable and Hilbert amplitude fields requiring similar computational time. Indeed, amplitude fields may need to be updated often in clinical practice. We obtain quick estimates of isostable amplitude fields by relaxing the convergence requirement of the isostable field. This enables us to use the faster Euler’s method to integrate trajectories. We select on a patient basis *n* (see equation (4)) as well as the time step to lower computation time as much as possible while avoiding major artefacts in the isostable amplitude field. We then approximately match on a patient basis the computation time taken to compute Hilbert amplitude fields by reducing the number of simulations and number of periods per simulations (the Euler-Maruyama method is already used). Single threaded computation times range from 19 to 454 s and are detailed in table B in the supplementary material together with the parameters used. The resulting amplitude fields are similar to full estimates (see figure B in the supplementary material). However the scale of isostable amplitude fields is not the same since the convergence requirement has been relaxed. Hilbert amplitude fields are noisier, with more missing values.

### 3.3 A phase space stimulation strategy drawing on amplitude fields

We describe a stimulation strategy that uses information from state space amplitude fields to decide whether it is more beneficial to stimulate now or at a later time of the ongoing period.

#### 3.3.1 Instantaneous and augmented response fields

**Figure 4:**
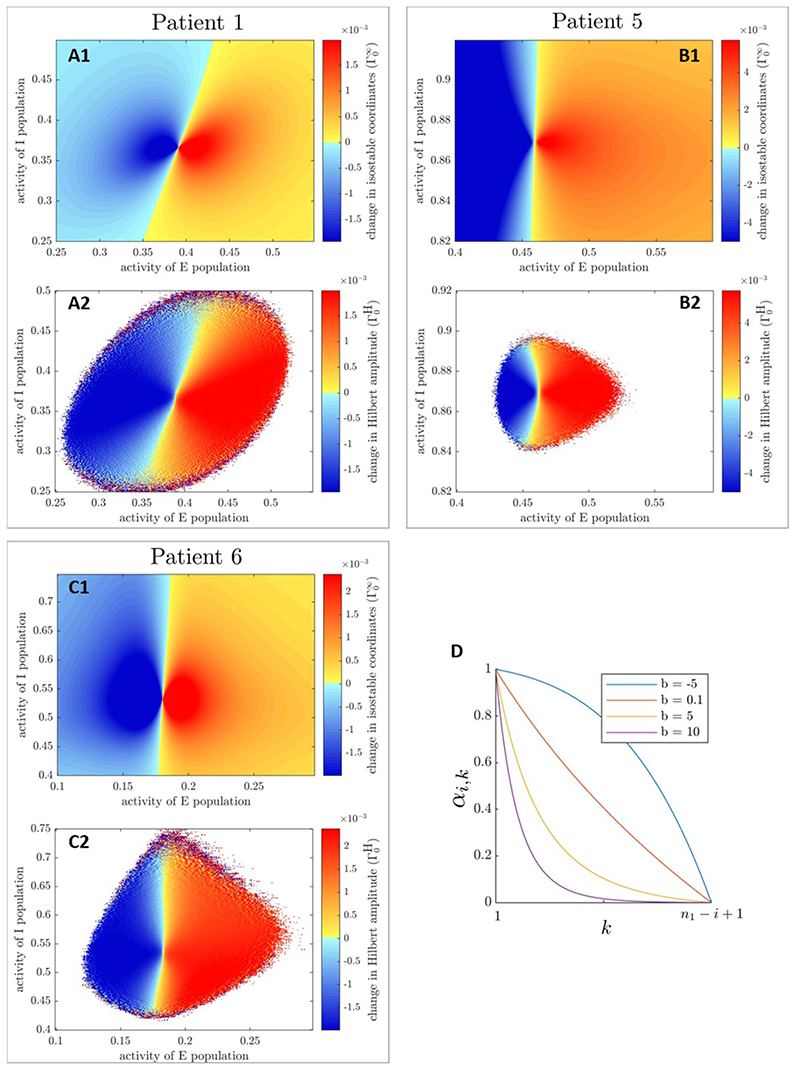
Instantaneous amplitude response fields for full estimates of asymptotic amplitude ($\Gamma_{0}^{\infty }$, panels (A1), (B1) and (C1)) and full estimates of mean- centered Hilbert amplitude ($\Gamma_{0}^{\text{H}}$, panels (A2), (B2), and (C2)) are shown for the three fitted WC models for $\delta X=[\begin{array}{c} \delta E_{0}\\ 0 \end{array}]$ (δ*E*_0_ are previously fitted stimulation magnitudes). White zones signify missing values. Negative values of the response fields (in blue) signify a decrease in amplitude (beneficial stimulation), positive values (in yellow to red) signify an increase in amplitude. Color scales are matched on a patient basis. Panel D illustrates the *k* — 1 step ahead discount factor α_*i,k*_ at time *t*_i_, for various values of the discounting parameter *b*. Time *t* *_n_1__* corresponds to the predicted end of the current period.

**Figure 5:**
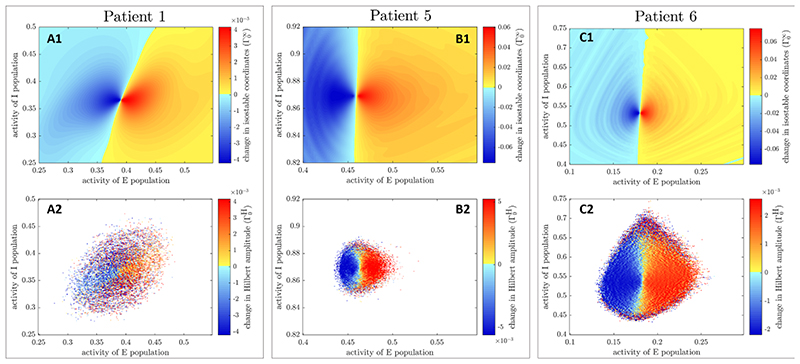
Instantaneous amplitude response fields for quick estimates of asymptotic amplitude $\Gamma_{0}^{\infty }$, panels (A1), (B1) and (C1)) and quick estimates of mean-centered Hilbert amplitude $\Gamma_{0}^{\text{H}}$, panels (A2), (B2), and (C2)) are shown for the three fitted WC models for $r\delta X=[\begin{array}{c} \delta E_{0}\\ 0 \end{array}]$ (δ*E*0 are previously fitted stimulation magnitudes). White zones signify missing values. Negative values of the response fields (in blue) signify a decrease in amplitude (beneficial stimulation), positive values (in yellow to red) signify an increase in amplitude. Color scales are only matched for patient 1 as field values are too different for other patients.

The starting point is a phase space amplitude field Ω(**X**) (we will be using isostable or Hilbert amplitude fields shown in figure 3, as well as in figures A and B in supplementary material). From there, we define the corresponding ‘instantaneous amplitude response field’ Γ_0_(**X**) = Ω(**X** + δ**X**) — Ω(**X**), where δ**X** is the stimulation vector. Instantaneous amplitude response fields for isostable amplitude $\left(\Gamma_{0}^{\infty }\right)$ and mean-centered Hilbert amplitude $\left(\Gamma_{0}^{\text{H}}\right)$ are presented in figure 4, panels (A)-(C). Zones where stimulation is beneficial correspond to negative values of the response fields (decrease in amplitude) and are shown in blue (the darker the blue, the greater the magnitude of the change in amplitude). Conversely, zones where stimulation is not beneficial correspond to positive values of the response fields (increase in amplitude) and are indicated with a colorscale from yellow to red. Instantaneous amplitude response fields for FP-centered Hilbert amplitude (which we denote $\Gamma_{0}^{{\text{H}}^{*}}$ ) are very similar to $\Gamma_{0}^{\text{H}}$ (see figure C in the supplementary material). Instantaneous amplitude response fields from quick estimates of Hilbert amplitude fields are more noisy (mean-centered Hilbert amplitude in the bottom row of figure 5, and FP- centered Hilbert amplitude in figure D in the supplementary material). Although quick estimates of isostable amplitude fields do not exhibit visible artefacts (top row of figure B in the supplementary material), patterns that were not present in figure 4 are seen in the instantaneous response fields corresponding to quick estimates of isostable amplitude fields. These patterns include deformations of constant field value contours (contrast the rightmost part of figure 4(A1) and of figure 5(A1)), as well as ripples organized in a concentric fashion (compare figures 4(C1)-5(C1)).

In what we call an ‘augmented instantaneous amplitude response field’ Γ(**X**, *t*), we embed the values of Γ_0_ on deterministic trajectories starting at **X** from *t* = 0 to *t* = *T*. The information will be used to inform the decision to stimulate or not at **X**. We are considering the (*E*, *I*) phase space, and the range of interest of *E* and *I* are discretized into *N_E_* and *N_I_* values, respectively. Algorithm 1 describes how we obtain Γ from Γ_0_ in discretized space and time. Stimulation is only beneficial when the amplitude response is negative (decrease in pathological signal amplitude, tremor amplitude). Positive values of the instantaneous amplitude response field are therefore of no interest to provide beneficial stimulation and are logged in Γ as zeros.

#### 3.3.2 Phase space stimulation using amplitude fields

 Based on an augmented instantaneous amplitude response field of choice, the decision to stimulate or not is made at each model integration time step using the current position in phase space (algorithm 2). Stimulation is provided as a single pulse, at maximum once per period according to the scheme. We track zero-crossings and estimate the zero-crossing phase (as detailed in section 2.4). To prevent instability when zero-crossing tracking fails, we enforce a maximum stimulation frequency (corresponding to *N*_lim_ time steps in algorithm 2) of 10 Hz, which is roughly twice the tremor frequency. The benefit of stimulating at later times of the ongoing period is estimated by the values stored in Γ in the space bin corresponding to the current position. The estimated values are based on deterministic trajectories,

**Algorithm 1. Obtain Γ**

      **for** (*i*, *j*) in {1,..., *N* *_E_*} × {1,..., *N_I_*} **do**

         simulate trajectories (*E*(*tk*),*I*(*tk*))_*k*∈{_1,...,*u*_}_

            with *t*_1_ = 0, *t_u_* = *T*, *E*(*t*_1_) = *E_i_*, and *I*(*t*_1_) = *Ij*.

         **for** *k* in {1,..., *u*} **do**

            find (*p*, *q*) ∈ {1,...,*N_E_*} × {1,...,*N_I_*} s.t.

            (*E_p_*,*I_q_*) is the closest to (*E*(*tk*),*I*(*tk*))

            **if** Γ_0_(*p*, *q*) < 0 and Γ_0_ (*p*, *q*) ⩽ Γ_0_ (*i*, *j*) **then**

                  Γ(*i*,*j*,*k*) ← Γ_0_ (*p*, *q*)

            **else**

                  Γ(*i*,*j*,*k*) ← 0

            **end if**

         **end for**

      **end for**

**Algorithm 2. Decide to stimulate at time step *ti***

      **if** no stimulation for *N*_lim_ time steps and no stimulation

            yet in current period **then**

         find *(p,q)* ∈ {1,...,*N*_*E*_} × {1,...,*N_i_*} s.t. (*E_p_*,*I_q_*)

            is the closest to (*E*(*ti*),*I*(*ti*))

         **if** Γ(*p*, *q*, 1) < 0 **then**

            **if** predicted period end already reached **then**

               stimulate

            **else**

               **if** $\Gamma (p,q,1)<\min_{k\in \{2,\ldots ,n_{1}-i+1\}}(\alpha_{i,k}\Gamma (p,q,k))$ **then**

                  stimulate

               **end if**

            **end if**

         **end if**

      **end if**

whereas the model includes Gaussian white noise. As a result, the actual benefit of waiting to stimulate is uncertain and will deviate from the average (noiseless) trajectory prediction, while the benefit of stimulating now is known exactly. Future estimates must therefore be discounted. From time *t_i_*, we define the *k —* 1 step ahead discount factor α*i*,*k* as

(5) $$\alpha_{i,k}=\left[\frac{1}{{\left(t_{i+k-1}-t_{n_{0}}\right)}^{b}}-\frac{1}{{\left(t_{n_{1}}-t_{n_{0}}\right)}^{b}}\right]\times {\left[\frac{1}{{\left(t_{i}-t_{n_{0}}\right)}^{b}}-\frac{1}{{\left(t_{n_{1}}-t_{n_{0}}\right)}^{b}}\right]}^{-1},$$

where *b* is the discounting parameter, *n_0_* is the index of the first time step of the current period, and *n_1_* is the index of the last time step of the current period. As shown in panel (D) of figure 4, α_*i*,*k*_ is one at *t_i_* (no discounting of the present value), and zero at the predicted end of the current period. It is also apparent that a larger *b* discounts more heavily future values. In algorithm 2, discount factors are not used if the end of the current period predicted by zero-crossing phase has been reached before the next zero-crossing is detected. In this case, stimulation is applied right then if beneficial.

### 3.4 Performance of phase space stimulation based on various amplitude fields

Using the three fitted patient models, we compare the performance of algorithm 2 when based on isostable amplitude, on mean-centered Hilbert amplitude, or on FP-centered Hilbert amplitude under three scenarios. As a reference, the performance of phase- locked stimulation (see section 2.4) is also reported for each scenario, and an efficiency comparison with HF stimulation is provided. We consider the cases of full estimates of the amplitude fields (see section 3.1) and of quick estimates of the amplitude fields (see section 3.2). We also investigate the influence of doubling the noise standard deviation *ζ* (for full estimates only). Under each scenario, we apply algorithm 2 based on $\Gamma_{0}^{\infty }$, on $\Gamma_{0}^{\text{H}}$, or on $\Gamma_{0}^{{\text{H}}^{*}}$ to the three fitted models for various stimulation magnitudes *δE* (given as multiples or fractions of δ*E*_0_), and for various values of the discounting parameter *b*. Hilbert amplitude instantaneous response fields have space bins with missing values (see for instance white zones in figure 4, panels (A2), (B2), and (C2)). Forfair comparison, the corresponding space bins are emptied in isostable amplitude instantaneous response fields. For compatibility with algorithm 2, Γ_0_ is set to zero in both cases for empty space bins, so that stimulation cannot be triggered at these locations. Smaller stimulation magnitude ratios δ*E*/δ*E*_0_ are used for patient 5 because of the comparatively small size of the Hilbert amplitude field available for this patient and the comparatively larger δ*E*_0_ (see table A in the supplementary material). To compare Hilbert and isostable amplitude stimulation as well as phase- locked stimulation, we measure stimulation efficacy by integrating the power spectrum density (PSD) of the *E* activity (which models the tremor signal). Efficacy is averaged using 30 trials of 5000 s of stimulation. Since repeated measures are performed on each patient, linear mixed effect models with random intercepts are used to assess statistical differences between phase space methods while accounting for within patient dependence. Specifically, we fit to each scenario the model

(6) power~method*stim*b+(1∣patient)

where method, stim, and *b* are the factors corresponding to the choice of amplitude field, the stimulation magnitude ratio δ*E*/δ*E*_0_, and the discounting parameter *b*, respectively. Following standard R notation, * indicates the inclusion of all main effects and interactions between the factors, and (1 |patient) indicates a random intercept per patient. We evaluate the significance of fixed effects using *F*-tests based on Satterthwaite’s method for denominator degrees-of-freedom [53]. Where the method factor is significant, post hoc comparisons between the three phase space methods are performed using *t*- tests with Bonferroni correction for robust multiple comparisons.

#### 3.4.1 Phase space stimulation based on full estimates of amplitude fields

**Figure 6:**
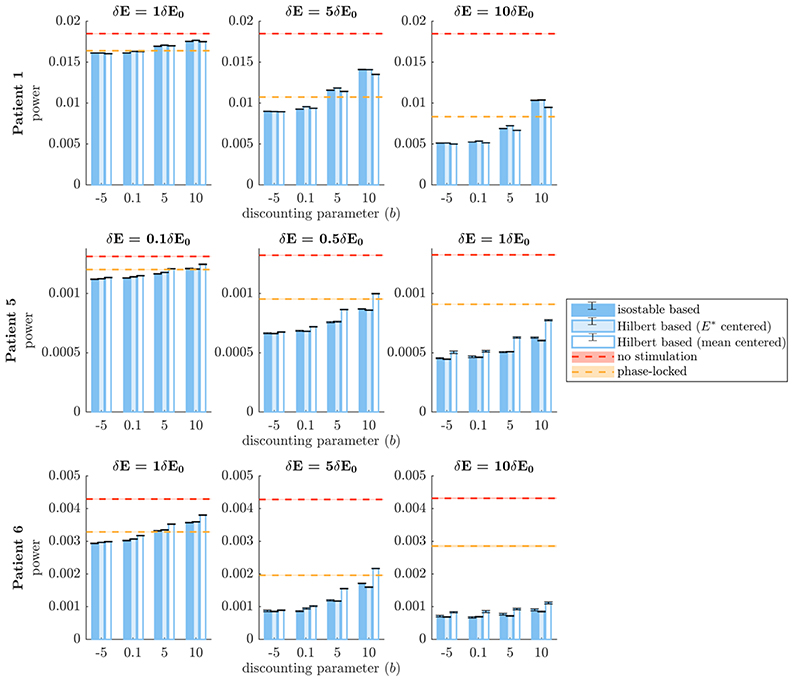
Comparison of the efficacy of phase space stimulation strategies based on full estimates of amplitude fields. Isostable amplitude fields correspond to darker blue bars, FP-centered Hilbert amplitude fields to lighter blue bars, and mean-centered Hilbert amplitude fields to white bars. Each row corresponds to a WC patient fit. Results are shown for a range of stimulation magnitude ratios δ*E*/δ*E*_0_ (increasing from left to right), and a range of discounting parameters *b*. The vertical axes represent efficacy measured as the integral of the PSD of *E* (which models the tremor signal). A no stimulation reference is indicated by red dashes, and phase-locked stimulation is indicated by orange dashes. Averages correspond to 30 trials of 5000 s of stimulation. SEM error bars are shown in black for Hilbert and isostable based stimulation and as shading for no stimulation and phase-locked stimulation.

**Figure 7:**
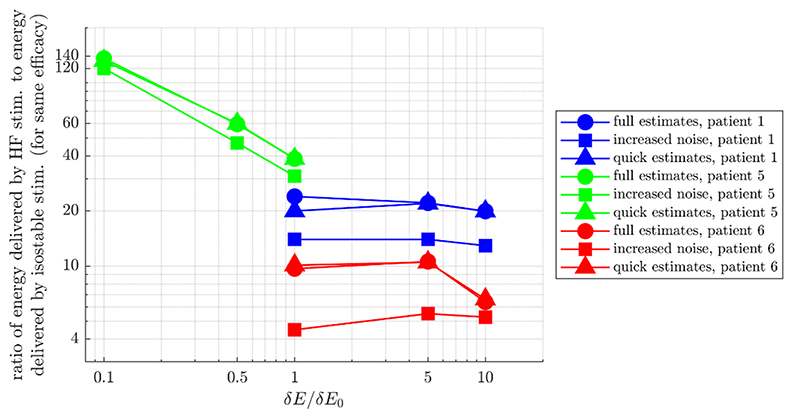
Comparison of the energy delivered to the thalamus for the same clinical effect by the best performing strategy based on isostable amplitude and by HF stimulation. The vertical axis represents the ratio of the energy delivered by HF stimulation to the energy delivered by phase space stimulation based on isostable amplitude (discounting parameter *b* = -5) for the same efficacy in the model. Efficacy is measured as the integral of the PSD of *E*, which models the tremor signal, and the efficacy match between stimulation types is within ±1%. The horizontal axis represents the increasing stimulation magnitude ratios δ*E*/δ*E*0 used for isostable stimulation. Each color corresponds to a WC patient fit, and each marker type to a comparison scenario (see sections 3.4.1–3.4.3).

Comparison of phase space stimulation based on amplitude field full estimates in figure 6 shows that stimulation based on isostable amplitude is mostly on par with stimulation based on Hilbert amplitude, and that phase space methods can be more effective than phase-locked stimulation. The method factor comparing phase space strategies was not significant overall in the corresponding mixed effect model (*p* = 0.0605, see table C in the supplementary material for more details). Post hoc comparisons were therefore not performed. The slight advantage of isostable amplitude for patients 5 and 6 for larger values of *b* compared to mean-centered Hilbert amplitude seems to be due to a more favorable centering of the isostable amplitude field. Indeed, the difference disappears for FP-centered Hilbert amplitude. Furthermore, we have confirmed that the difference between $\Gamma_{0}^{\text{H}}$ and $\Gamma_{0}^{{\text{H}}^{*}}$ can be reproduced simply by shifting $\Gamma_{0}^{{\text{H}}^{*}}$ along the *E* dimension (see figure E in the supplementary material). Despite similar performance under this scenario, there are still non negligible differences between $\Gamma_{0}^{{\text{H}}^{*}}$ and $\Gamma_{0}^{\infty }$ as evidenced by figure F in the supplementary material. Additionally, we note that our phase space stimulation strategies based on amplitude fields are more effective than phase-locked stimulation for low discounting parameters and high stimulation magnitudes. To match the efficacy of stimulation based on isostable amplitude at *b* = –5, open-loop HF stimulation has to deliver from 4.5 times to 120 times more energy to the thalamus (see round markers in figure 7). This highlights the greater energy efficiency of phase space stimulation based on amplitude fields and its potential for a lower occurrence of side-effects compared to HF stimulation.

#### 3.4.2 Robustness to noise

**Figure 8:**
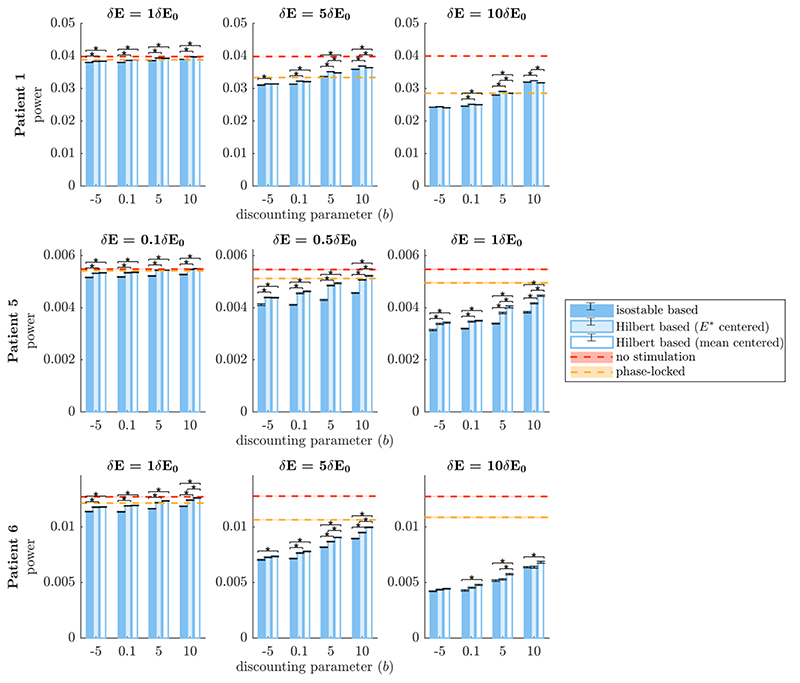
Comparison ofthe efficacy of phase space stimulation strategies based on full estimates ofamplitude fields when doubling the noise intensity. Isostable amplitude fields correspond to darker blue bars, FP-centered Hilbert amplitude fields to lighter blue bars, and mean-centered Hilbert amplitude fields to white bars. Each row corresponds to a WC patient fit. Results are shown for a range of stimulation magnitude ratios δ*E*/δ*E*_0_ (increasing from left to right), and a range of discounting parameters *b*. The vertical axes represent efficacy measured as the integral of the PSD of *E* (which models the tremor signal). A no stimulation reference is indicated by red dashes, and phase-locked stimulation is indicated by orange dashes. Averages correspond to 30 trials of 5000 s of stimulation. SEM error bars are shown in black for Hilbert and isostable based stimulation and as shading for no stimulation and phase-locked stimulation. Black stars denote significant differences under Bonferroni correction.

Robustness to noise was evaluated by doubling the noise standard deviation in patient models. PSDs of model simulations for the three fitted patients are shown in the baseline and increased noise conditions in figure G in the supplementary material. The main PSD peak width is substantially enlarged in the increased noise condition, in particular for patients 5 and 6. In this scenario using full estimates of amplitude fields, the method factor of the corresponding mixed effect model was significant (*p* < 10^–15^, see table C in the supplementary material for more details). Significance of post-hoc comparisons under Bonferroni correction is indicated by black stars in figure 8. There is an overall reduction in efficacy of all stimulation strategies compared to the baseline noise condition (figure 6), including for phase-locked stimulation. As before, phase space stimulation strategies based on amplitude fields are more effective than phase-locked stimulation for *b =* –5 in all the cases shown in figure 8 (although the difference is small for the lowest stimulation case in patient 1), and for a broader range of discounting parameters at high stimulation magnitude. Among phase space stimulation strategies, stimulation based on isostable amplitude outperforms stimulation based on Hilbert amplitude in a number of cases (patient 5, and in most conditions investigated for patient 6). However these differences are small, and are even smaller or not favorable for patient 1. When the noise standard deviation is larger, trajectories cover more of phase space. Because Hilbert amplitude fields rely on averaging, zones closer to the edges of Hilbert amplitude fields are noisier as trajectories of large amplitude are more sparse. Isostable amplitude fields are immune to this issue, which may explain the slightly better robustness of isostable based stimulation to an increase in the noise standard deviation. Despite the increased noise level, stimulation based on amplitude fields is still more efficient than open-loop HF stimulation. To match the efficacy of stimulation based on isostable amplitude at *b =* –5, open-loop HF stimulation has to deliver from 4.5 times to 120 times more energy to the thalamus (see square markers in figure 7).

#### 3.4.3 Phase space stimulation based on quick estimates of amplitude fields

**Figure 9:**
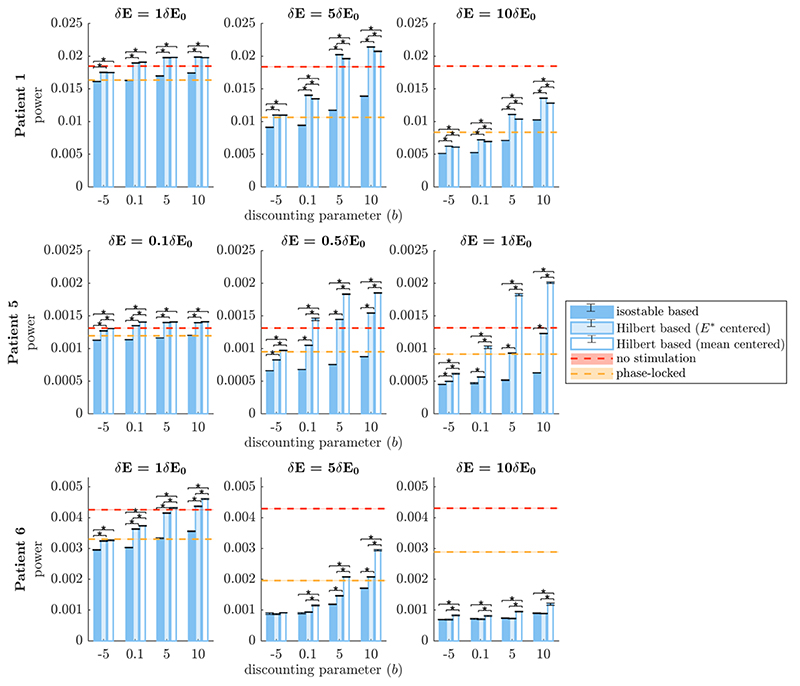
Comparison of the efficacy of phase space stimulation strategies based on quick estimates of amplitude fields. Isostable amplitude fields correspond to darker blue bars, FP-centered Hilbert amplitude fields to lighter blue bars, and mean-centered Hilbert amplitude fields to white bars. Each row corresponds to a WC patient fit. Results are shown for a range of stimulation magnitude ratios δ*E*/δ*E*_0_ (increasing from left to right), and a range of discounting parameters *b*. The vertical axes represent efficacy measured as the integral of the PSD of *E* (which models the tremor signal). A no stimulation reference is indicated by red dashes, and phase-locked stimulation is indicated by orange dashes. Averages correspond to 30 trials of 5000 s of stimulation. SEM error bars are shown in black for Hilbert and isostable based stimulation and as shading for no stimulation and phase-locked stimulation. Black stars denote significant differences under Bonferroni correction.

In clinical practice, full estimates of amplitude fields are unlikely to be useful because of their computational cost (on the order of hours). The performance of stimulation strategies based on quick estimates of amplitude fields which can be computed in minutes is compared in figure 5. In this scenario, the method factor of the corresponding mixed effect model was found significant (*p* < 10^–15^, see table C in the supplementary material for more details). Isostable amplitude based stimulation offers a clearer advantage compared to Hilbert amplitude based stimulation (see post-hoc comparison under Bonferroni correction in figure 8) than in previous scenarios. While instantaneous amplitude response fields based on quick estimates of isostable amplitude exhibit some artefacts (see top panels in figure 5), these artefacts do not seem to notably affect the performance of isostable based stimulation. In fact, the efficacy of phase space stimulation based on quick estimates of isostable amplitude and the efficacy of phase space stimulation based on full estimates of the isostable amplitude are similar (compare the darker blue bars in figures 6 and 9). In contrast, the increased noise in instantaneous amplitude response fields based on quick estimates of Hilbert amplitude (see bottom panels in figure 5, as well as figure D in the supplementary material) is responsible for the decreased performance of Hilbert amplitude based stimulation. Even when isostable amplitude fields are computed in minutes, isostable amplitude based stimulation (and in fewer cases Hilbert amplitude based stimulation) is as before more effective than phase-locked stimulation, in particular for low discounting values (with the exception of patient 1 at the lowest stimulation level, where performance is similar). Additionally, stimulation based on isostable amplitude fields computed in minutes is still more efficient than open-loop HF stimulation, requiring from 6.6 times to 132 times less energy to be delivered to the thalamus (see triangular markers in figure 7).

## 4 Discussion

In this study, we showed that phase space stimulation based on isostable or Hilbert amplitude fields can be more effective than phase-locked stimulation and is more efficient than open-loop HF stimulation, even when noise is increased from the baseline noise level fitted to patients. Stimulation based on isostable amplitude was in most cases on par with stimulation based on Hilbert amplitude, with slight favorable differences accounted for by a more accurate centering of isostable amplitude fields. The performance of isostable amplitude based stimulation and its advantage over phase-locked stimulation and HF stimulation was maintained when using quick estimates of amplitude fields obtained in minutes, which is likely to be a clinical requirement. In contrast, the performance of stimulation based on quick estimates of

Hilbert amplitude fields significantly degraded due to noisier estimates. This suggests that there might be a benefit to using more broadly amplitude fields, in particular isostable amplitude fields, for modelbased optimization of stimulation in neurological disorders.

### 4.1 Isostable amplitude

Although isostables have been developed with model reduction in mind [37–39], there are similarities between isostable amplitude and Hilbert amplitude, which is one of the preferred measures of synchrony in neurophysiological data. Trajectories starting from the same isostable converge synchronously to the attractor, i.e. reach subsequent isostables at the same time. In our (*E*, *I*) model, trajectories starting from the same isostable share the same Hilbert amplitude (true regardless of whether the Hilbert amplitude is defined based on *E* or on *I*). This is reflected by the fact that isostable and Hilbert amplitude fields are strikingly similar (see figure 3). Although amplitude values and gradients are different, contours of equal amplitude have very similar shapes. In ℝ^2^, a relationship between isostable and Hilbert amplitude might be revealed by studying the approximation of isostable coordinates given by equation (4). This equation can be interpreted as the norm of a 2D vector, similarly to the Hilbert amplitude. The Hilbert amplitude is indeed the norm of the vector formed by the real and the imaginary parts of the analytic signal.

In dynamical systems, phase-amplitude reductions such as the isochron-isostable reduction are augmentations of classical phase reductions, and various techniques are available to define an amplitude coordinate (see [54] for a review). In contrast with methods based on isostable amplitude, the euclidean distance to the attractor is used as an amplitude variable in [55], and a transverse variable obtained from parametrizing isochrons is used in [56]. These methods are applicable when the attractor is a limit cycle, which is not the case in the present work. Benefits of the isostable method chosen [37] include its computational efficiency, and its truly global character. Isotables can be computed everywhere in the basin of attraction of the FP without limitations. The theory behind isostables considers purely deterministic dynamical systems, whereas the fitted WC models employed in our study involve Gaussian white noise. Theories have been developed to account for the effect of noise in isochrons [57–59], but isostables are lagging behind in this respect. However, isostable amplitude fields share similarities with Hilbert amplitude fields which were obtained by averaging noisy trajectories. It therefore seems likely that isostable amplitude contours would not change substantially were Gaussian white noise explicitly accounted for.

Control strategies developed in [18–20] are based on the knowledge of individual oscillator phase response curves, likely challenging to obtain in patients. In [21], phase synchronization of neural populations is directly considered. However the amplitude response of individual populations, which may be of importance at the mesoscale level, is not taken into account. Instead of considering phase synchronization of oscillators, we directly focus on amplitude response at the population level, which is more directly related to symptoms.

### 4.2 Comparing phase space stimulation strategies based on isostable and Hilbert amplitude

The closed-loop phase space stimulation strategy suggested in section 3.3 produces overall similar results when based on full estimates of isostable amplitude and when based on full estimates of Hilbert amplitude. Minimizing isostable amplitude is minimizing convergence time to the attractor. Knowledge of the future evolution of the (deterministic) non-linear system embedded in isostable coordinates may theoretically provide an advantage in controlling the Hilbert amplitude of the stochastic system. Instantaneous response fields obtained from isostable amplitude are in fact different from their Hilbert amplitude counterparts, even when FP-centered Hilbert amplitude is used (see figure F in the supplementary material). However convergence time information captured by isostable amplitude does not seem to directly translate into major differences in performance with Hilbert amplitude based stimulation in the two-dimensional models studied (see figure 6). The benefit of using isostable amplitude fields in two-dimensional models may reside instead in the ability to obtain quick estimates of amplitude maps more reliably than with Hilbert amplitude. This is reflected in the better relative performance of isostable amplitude based stimulation in figure 9. However Hilbert amplitude based stimulation is still competitive in some cases, such as for low discounting parameter and high stimulation for patients 5 and 6. It is worth emphasizing that the resolution and coverage that can obtained for isostable amplitude fields is to our best knowledge out of reach for Hilbert amplitude fields. To obtain Hilbert amplitude fields, considering noisy trajectories is necessary to explore more of phase space, which drastically limits the resolution that can be obtained for a given number of simulations. To level the playing field for our comparison, isostable amplitude fields were masked to match the coverage of Hilbert amplitude fields, and the same resolution was used for both amplitude fields.

Differences in where stimulation is provided in phase space based on isostable or Hilbert amplitude fields are highlighted in figures H, I, and J in the supplementary material. Stimulation based on isostable amplitude tends to happen a little later in phase, although this effect is diminished when comparing isostable amplitude to FP-centered Hilbert amplitude instead of mean-centered Hilbert amplitude (see for instance panel (A) and (B) in figure I). Isostable amplitude based stimulation also tends to be more focal in most cases. This may be due to the fact that Hilbert amplitude fields are not as smooth (see figure 3) since they are obtained by averaging stochastic trajectories. For quick estimates, because stimulation maps based on Hilbert amplitude are not as reliable as for full estimates, stimulation is more likely to be delivered in unfavorable phase space locations as seen in (C) panels in figures H–J (supplementary material). Finally, isostable and Hilbert amplitude response fields do not exhibit the same boundaries between regions where stimulation is beneficial and regions where it is not. This is particularly true in patients 5 and 6 (see for instance figure 4, panels (A)–(C)).

### 4.3 Limitations of the study

An important limitation of this study is that because only three cases with a statistically significant phase response were available, our comparison is based on these three cases only. Because of experimental difficulties, patient recordings are short, and nonsignificant patients might simply have required more data. However essential tremor is an heterogeneous disorder, and it might also be the case that certain patient subtypes respond better to phase-locked stimulation. Nevertheless, the three patient models studied are heterogeneous as shown by notably different amplitude fields from one patient to another (figure 3), suggesting that phase space stimulation based on amplitude fields may be applicable to various patient subtypes.

While the WC model is useful to model DBS in ET, it is worth discussing its limitations in the context of the literature. The central origin of ET is well established (see e.g. [1, 60, 61]). In particular, thalamic activity has been associated with activity in peripheral muscles driving the tremor [2–4], and some ET models are exclusively based on biophys- ically detailed representations of thalamic neurons [62, 63]. Other models include the broader cerebello- thalamo-cortical network [51, 64, 65]. Since detailed knowledge of the central mechanism of generation of ET is not available, mean-field models are natural candidates to study ET [20, 32, 51]. The WC model used in this study is a heuristically derived mean-field model [47]. As such, it benefits from a low number of parameters while retaining some level of description of a microscopic biological reality. However, bursting of thalamic neurons is not described by the WC model and might play a role in ET [51]. Another limitation is that our WC model portrays a central feedback loop, and does not account for the interaction of the centrally generated component of ET with the mechanical stretch reflex, whose involvement may depend on the disease state [66, 67]. The mesoscopic scale of the WC model makes it particularly suited to be fitted to mesoscopic data such as local field potential recordings obtained from DBS electrodes, or tremor recordings (as used in [32]). It is likely that more complex models with more parameters would overfit such data, and would be too computationally demanding to fit in practice. Despite the limitations mentioned above, the WC model has been shown to be adept at reproducing the effects of high-frequency DBS in ET [51] and PD [52], as well as the phase-dependent effects of phase-locked DBS in ET in datasets with statistically significant response curves [32]. Additionally, our phase space stimulation strategies based on amplitude fields can be applied to more complex models than the WC model. The isostable computation method described in section 2.2 is limited to two-dimensional FP dynamics, but the method can be extended to higher dimensions.

The requirement to fit a model to patient data to provide stimulation in phase space adds complexity. To inform stimulation using the Hilbert transform without relying on models, data trajectories could directly be portrayed in the (*y*,*H*(*y*)) space, where *y* is the centered tremor signal. However, in the case of ET, patients are asked to assume a tremor provoking posture to measure the response to phase-locked DBS [10]. Because patients fatigue quickly, only short recordings can be obtained (on the order of 10 min [46]). In these short datasets, the dependence of phase-locked DBS effects on the Hilbert phase of stimulation can be estimated in some patients [10, 32] (12 phase bins). The dependence of the effects of stimulation on the Hilbert amplitude of the on-going tremor is more difficult to measure (three amplitude bins with large error bars in [20]). An instantaneous amplitude response field in the (*y*, H (*y*)) space detailed enough to optimize stimulation is therefore out of reach. Moreover, providing stimulation according to the Hilbert phase of tremor alone is unlikely to bring optimal benefits.

An algorithm learning a target phase and a power threshold online to stimulate according to phase and power such as in [22] has many advantages, in particular its simplicity and model-free character. However, the response to stimulation is measured in [22] on a short timescale, and the subtle effects of phase- locked stimulation seen in patient data might therefore be overlooked. Moreover, stimulating according to a target phase and a single power threshold may be suboptimal compared to stimulation in phase space based on amplitude fields, where the decision to stimulate depends on the state, and predictions about the future evolution of the state can be taken into account. How the strategies would compare in practice is however unclear, in particular in the light of the greater complexity of phase space strategies based on amplitude fields, which require model fitting.

Given model parameters, a corresponding augmented instantaneous amplitude response field (defined in section 3.3.1), and the state, our phase space stimulation strategy based on amplitude fields can run very quickly (real time is likely to be achievable). While the model state would need to be estimated in near real time, we expect model parameters to change more slowly. Model parameters would therefore be updated and the amplitude fields (and corresponding response fields) computed on this slower time scale, which may be on the order of minutes. This is compatible with our results based on quick estimates of amplitude fields (see table B in the supplementary material for single threaded computation time of quick estimates). Estimation of model parameters and state may be achievable using unscented Kalman filtering as done in [25] for a spatially extended WC model. However the operational practicality of phase space stimulation strategies based on amplitude fields needs to be further evaluated.

### 4.4 Future directions

Amplitude field based stimulation for neurological disorders could be investigated further. In PD, motor symptoms are correlated with increased subthalamic nucleus (STN) beta band oscillatory power [68–72]. Excessive synchrony in PD has been explored with isostables in a model of coupled neurons [43], but not in a model constrained with experimental data. The WC model can describe the feedback loop composed of the STN and the globus pallidus pars externa [73–76], which plays a role in PD excessive beta synchrony. Similarly to what was done in the present study, amplitude field based stimulation could be applied to WC focus models fitted to PD patients, and compared to simpler stimulation strategies. Moreover, because isostables are related to convergence properties of a system, isostable amplitude could be provided as an additional feature to complement Hilbert amplitude when trying to optimize stimulation with machine learning approaches. Furthermore, isostable coordinates are at present limited to situations where a model is available. Obtaining the Koopman operator from data is being researched [77–79], which could make possible to recover isostables from data as there is a strong connection between the Koopman operator and isostables [37]. A method to directly obtain isostables from data has very recently been suggested [80]. However it relies on limit cycle dynamics, and it is also unknown whether the technique could be robust to the large levels of noise seen in neurophysiological data. Finally, convergence time information embedded in isostable amplitude fields may prove more profitable to control more complex, higher dimensional models, such as mean-field models of coupled populations of theta neurons [81, 82]. In higher dimensions, the computational advantage of isostable amplitude fields compared to Hilbert amplitude fields should also be larger.

## 5 Conclusion

Although isostable amplitude has been successfully applied to control the state of various systems, it has not been tested on patient models in the context of optimizing DBS, and has not been compared to standard definitions of amplitude. Using models fitted to ET patient data, we showed that amplitude field based phase space stimulation strategies may be beneficial compared to phase-locked stimulation and open-loop HF stimulation. Additionally, given limited computation time, phase space stimulation based on isostable amplitude may be more effective than phase space stimulation based on Hilbert amplitude. Our study opens opportunities for model-based control of pathological oscillations in neurological disorders.

## Supplementary Material

Supplementary material

## Data Availability

The datasets analyzed in the current study are available online [46]. We are sharing code at https://github.com/benoit-du/amplitude Fields to compute the isostable and Hilbert amplitude fields associated with the basin of attraction of two-dimensional dynamical systems with complex conjugate eigenvalues.
