# Supplementary material for: Optimizing deep brain stimulation based on isostable amplitude in essential tremor patient models
Source: J Neural Eng. Author manuscript; Available in PMC 2021 May 4. (PMC7610712; doi:10.1088/1741-2552/abd90d)
Supplement: Supplementary material [file EMS121797-supplement-Supplementary_material.pdf]

Supplementary material

Parameter	Symbol	Best fit values		
		Patient 1	Patient 5	Patient 6
Stimulation magnitude	δE_0	0.001684	0.00598	0.001686

Table A: Stimulation magnitude values obtained from data fits in [32].

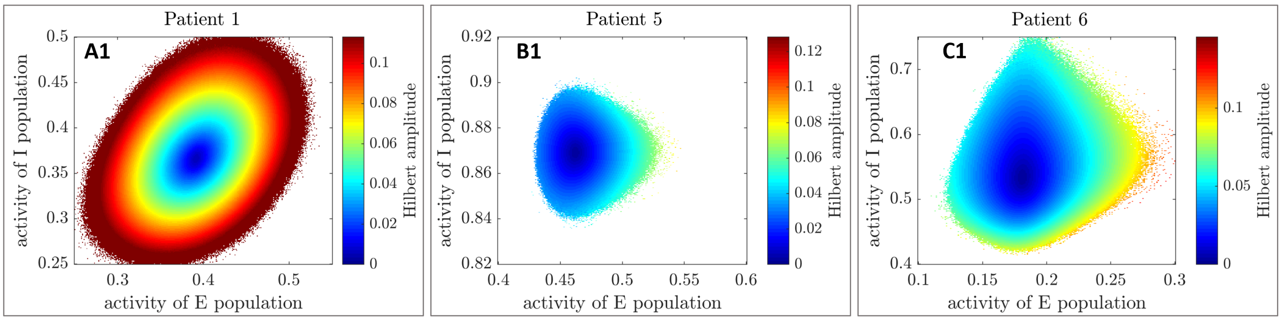

Figure A: FP-centered Hilbert amplitude fields for the three WC patient fits. White zones signify missing values.

		Patient 1	Patient 5	Patient 6
isostable amplitude fields	number of periods (n)	5	20	60
	integration time step	0.001	0.001	0.0004
	computation time (s)	19	92	430
Hilbert amplitude fields	number of simulations	30	50	125
	number of periods per simulation	35	50	125
	integration time step	0.001	0.001	0.001
	computation time (s)	37	109	454

Table B: Parameters and single threaded computation times to obtain quick estimates of isostable and Hilbert amplitude fields. The number of periods n enters isostable computation in equation (4). Both versions of Hilbert amplitude fields (mean-centered and FP-centered) use the same parameters, and the computation times are very similar for a given patient (values reported are for mean-centered Hilbert amplitude fields).

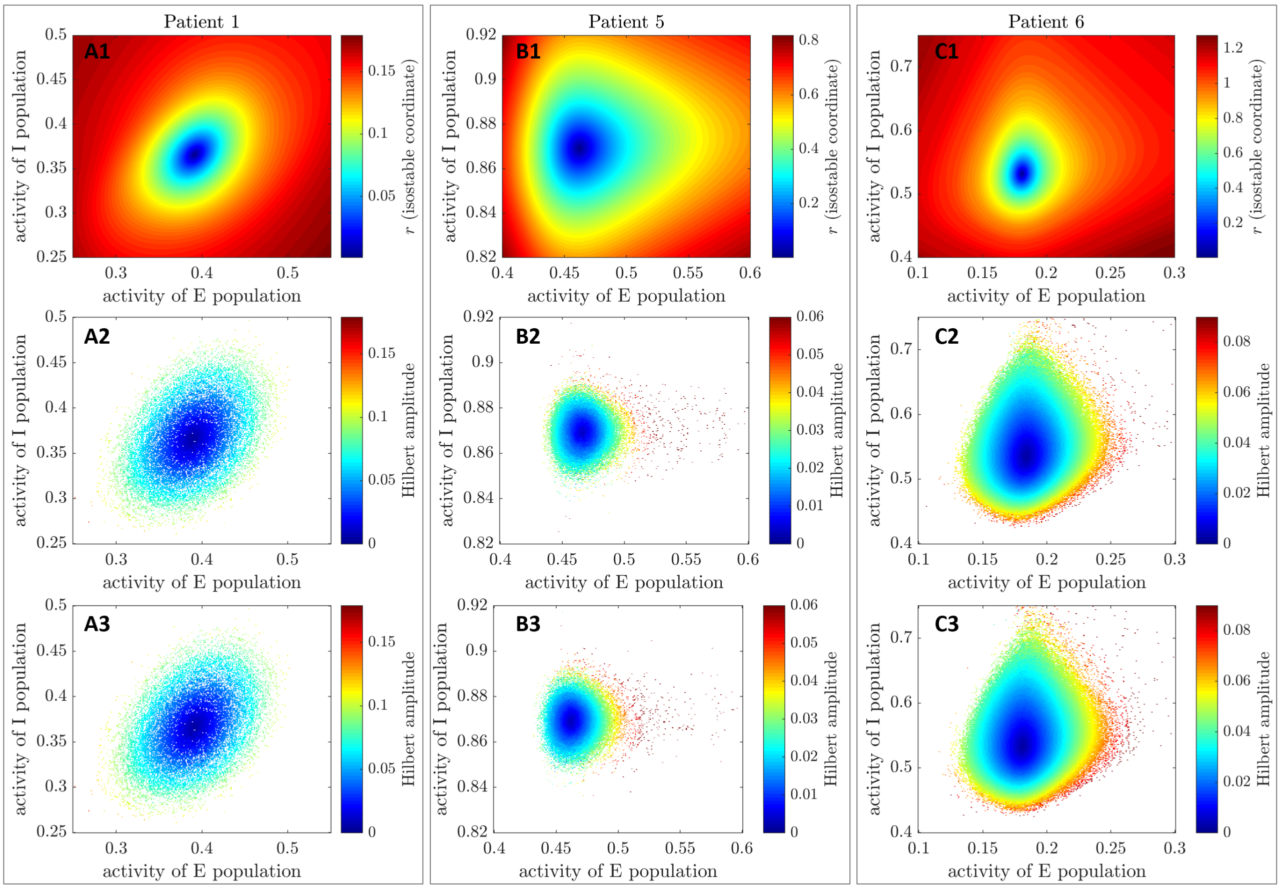

Figure B: Quick estimates of isostable amplitude field (top row), mean-centered Hilbert amplitude fields (middle row), and FP-centered Hilbert amplitude fields (bottom row) for the three WC patient fits. Each column corresponds to a patient fit. White zones signify missing values. Colour scales are only matched between isostable and Hilbert amplitude fields for patient 1.

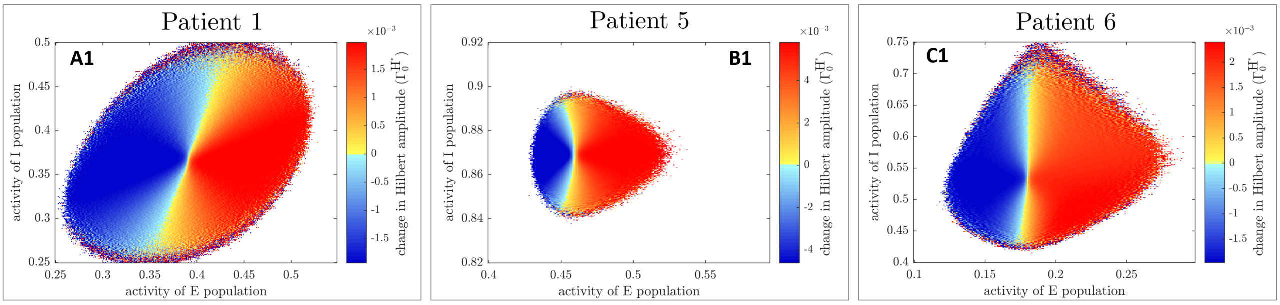

Figure C: Instantaneous amplitude response fields for full estimates of FP-centered Hilbert amplitude ($\Gamma_0^{H^*}$) are shown for the three fitted WC models for $\delta\mathbf{X} = \left[\begin{smallmatrix}\delta E_0\\0\end{smallmatrix}\right]$ (δE_0 are previously fitted stimulation magnitudes). White zones signify missing values. Negative values of the response fields (in blue) signify a decrease in amplitude (beneficial stimulation), positive values (in yellow to red) signify an increase in amplitude.

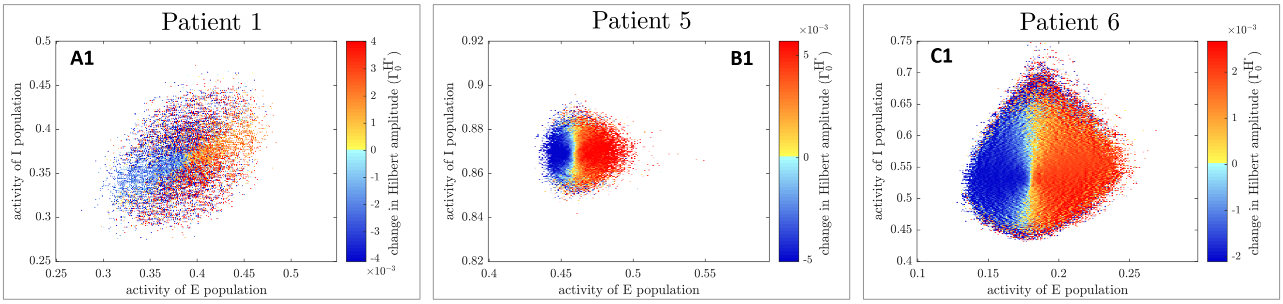

Figure D: Instantaneous amplitude response fields for quick estimates of FP-centered Hilbert amplitude ($\Gamma_0^{H^*}$) are shown for the three fitted WC models for $\delta\mathbf{X} = \begin{bmatrix} \delta E_0 \\ 0 \end{bmatrix}$ (δE_0 are previously fitted stimulation magnitudes). White zones signify missing values. Negative values of the response fields (in blue) signify a decrease in amplitude (beneficial stimulation), positive values (in yellow to red) signify an increase in amplitude.

	stim	b	method	stim*b	stim*method	b*method	stim*b*method
full estimates	$< 10^{-15}$	$< 10^{-15}$	0.0605	$5.31{\times}10^{-12}$	0.258	0.967	0.999
elevated noise	$< 10^{-15}$	$< 10^{-15}$	$< 10^{-15}$	$< 10^{-15}$	0.052	0.659	0.996
quick estimates	$< 10^{-15}$	$< 10^{-15}$	$< 10^{-15}$	$1.73{\times}10^{-15}$	$1.50{\times}10^{-7}$	$6.40{\times}10^{-7}$	0.0387

Table C: P-values associated with fixed effects included in the linear mixed effect models used to compare amplitude field based stimulation methods. The first row corresponds to the model fitted under the scenario using full estimates of amplitude field and the baseline noise level. The second row corresponds to the model fitted under the scenario using full estimates of amplitude field and twice the baseline noise level. The third row corresponds to the model fitted under the scenario using quick estimates of amplitude field and the baseline noise level. P-values correspond to F-tests using Satterthwaite's method for denominator degrees-of-freedom. P-values lower than 5% are highlighted in green.

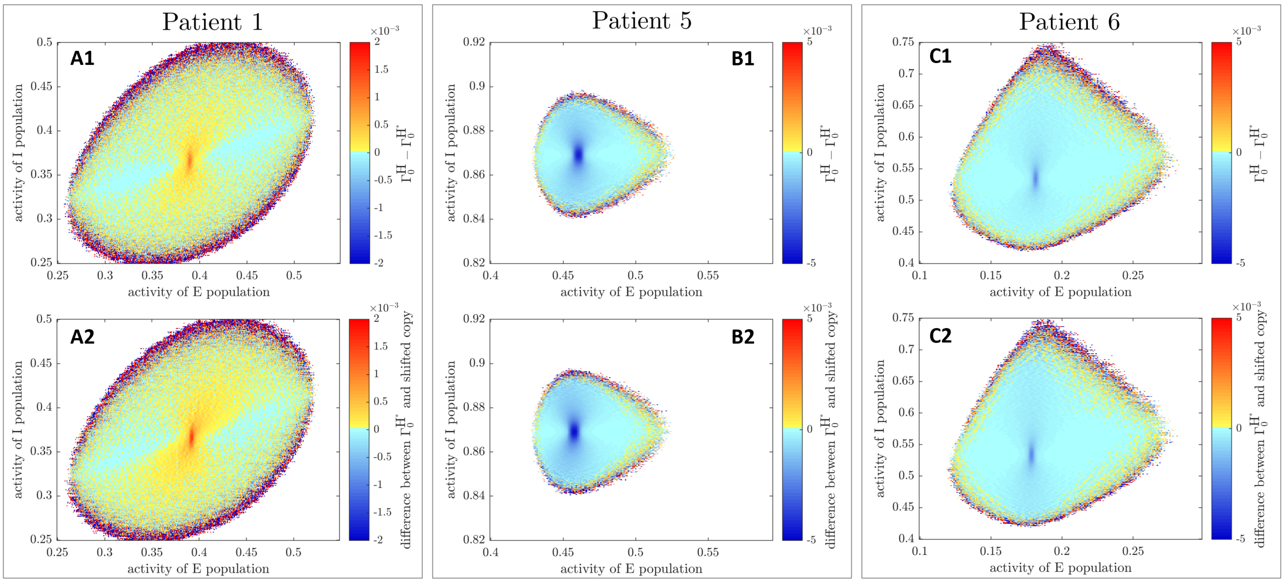

Figure E: Fields representing the differences between instantaneous amplitude response fields for mean-centered Hilbert amplitude (Γ_0^{H}) and for FP-centered Hilbert amplitude ($\Gamma_0^{\mathrm{H}^*}$) are shown in the top row for the three fitted WC models. In the bottom row, a copy of $\Gamma_0^{\mathrm{H}^*}$ shifted along the E direction is subtracted from $\Gamma_0^{\mathrm{H}^*}$. The similarities between the top and bottom rows for a given patient suggest that the main difference between Γ_0^{H} and $\Gamma_0^{\mathrm{H}^*}$ is a shift along the E direction. The shift is $1/120^{\mathrm{th}}$ of the E range shown in the plot for A2, and $-1/60^{\mathrm{th}}$ for B2 and C2. The stimulation magnitude is the fitted stimulation magnitude. White zones signify missing values.

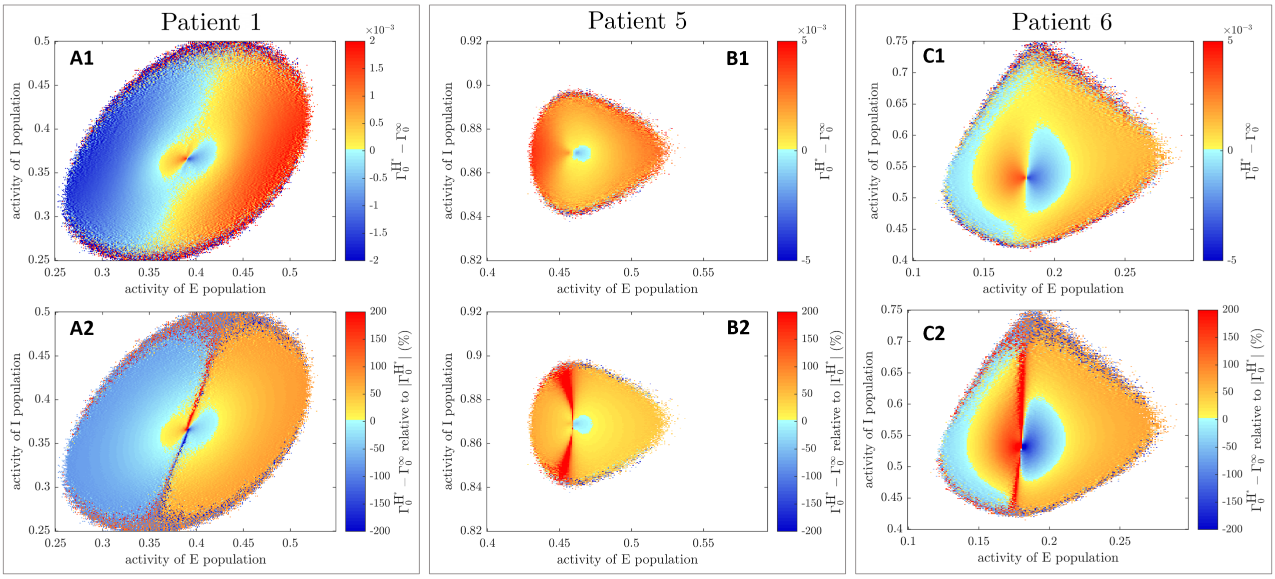

Figure F: Differences between instantaneous amplitude response fields for FP-centered Hilbert amplitude ($\Gamma_0^{\mathrm{H}^*}$) and asymptotic amplitude (Γ_0^{∞}) are shown for the three fitted WC models. The absolute difference is shown in the top row, and the bottom row presents the difference relative to ($|\Gamma_0^{\mathrm{H}^*}|$). The stimulation magnitude is the fitted stimulation magnitude. White zones signify missing values.

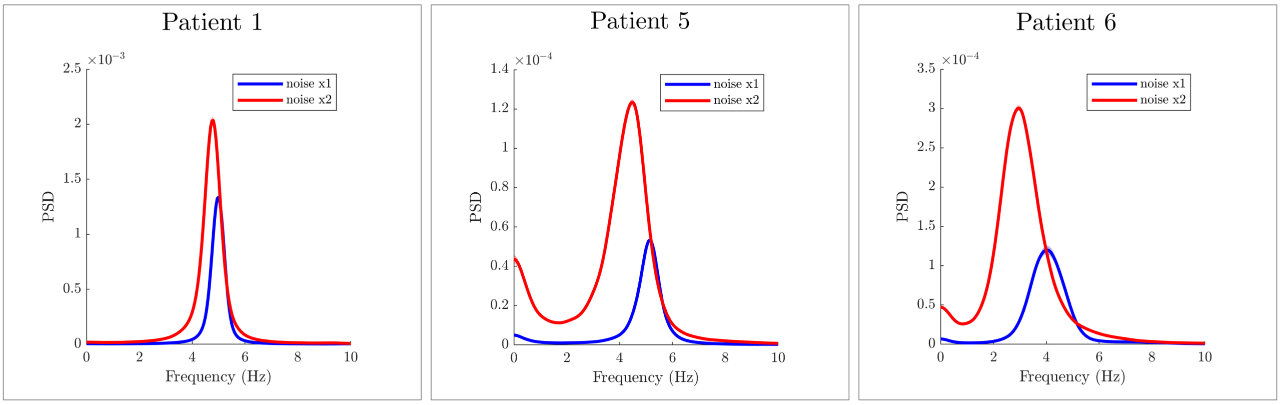

Figure G: Power spectra of simulated patients models using the fitted noise intensity (blue) and twice the fitted noise intensity (red). Shaded SEM error bars are shown in blue and red, respectively. The spectra were obtained from five trials of 1000 s.

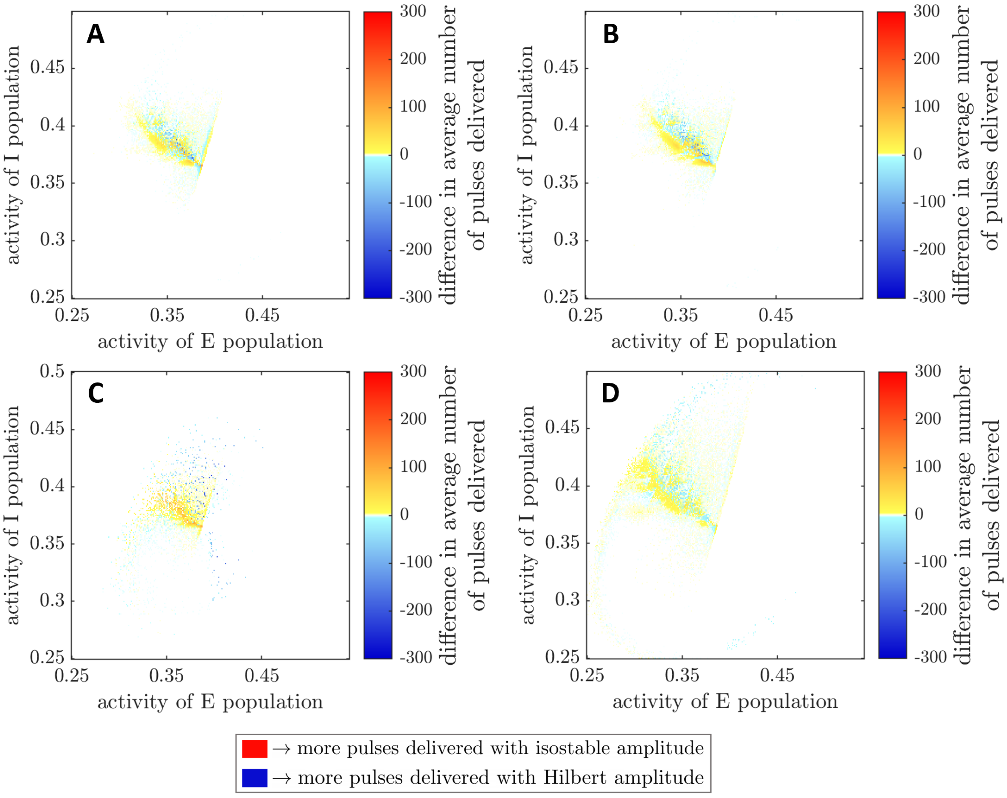

Figure H: Comparison of the average number of pulses delivered by phase space stimulation based on isostable amplitude and on Hilbert amplitude for patient 1, $b = 0.1$, $\delta E = 5\delta E_0$. Panel A and B correspond to full estimates of amplitude fields and baseline noise level. The Hilbert amplitude field used is mean-centered in panel A, and FP-centered in panel B. Panel C corresponds to quick estimates of amplitude fields and baseline noise level. Panel D corresponds to full estimates of amplitude fields and twice the baseline noise level. Panel C and D are based on a mean-centered Hilbert amplitude field. The color scale refers to differences in the average number of pulses delivered between stimulation based on isostable amplitude and on Hilbert amplitude. A positive difference (yellow to red) means that more pulses were delivered with the strategy based on isostable amplitude at this location. Results in each panels were obtained with three trials of 5000 s of stimulation.

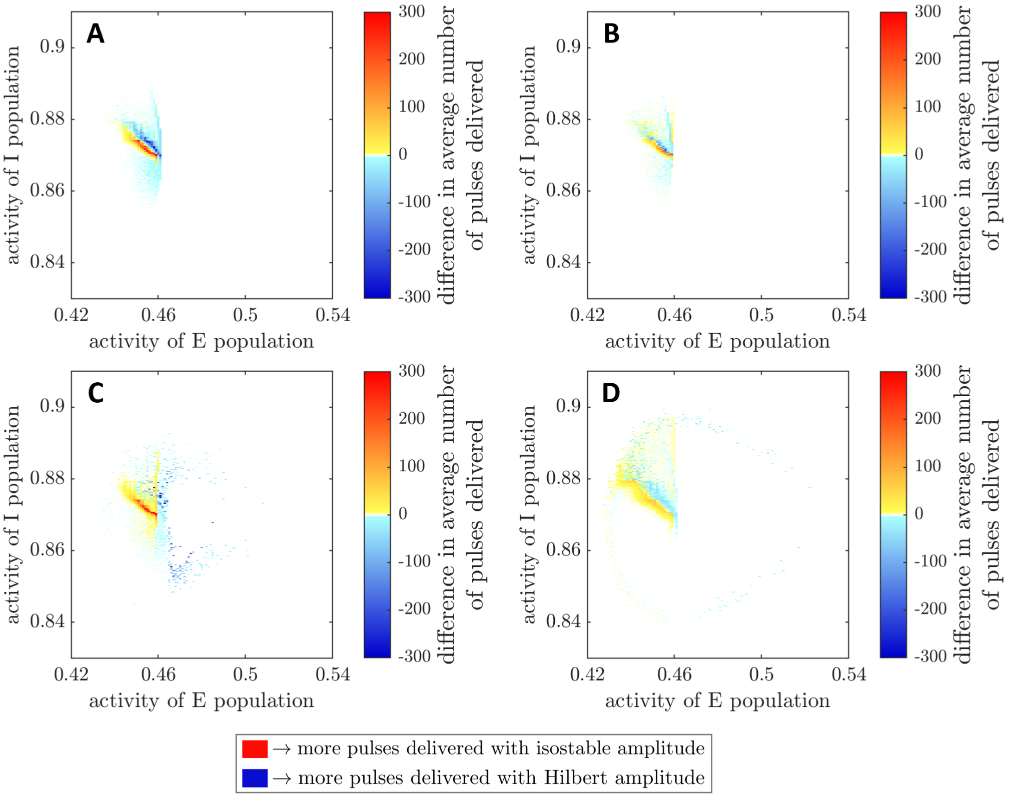

Figure I: Comparison of the average number of pulses delivered by phase space stimulation based on isostable amplitude and on Hilbert amplitude for patient 5, $b = 5$, $\delta E = \delta E_0$. Panel A and B correspond to full estimates of amplitude fields and baseline noise level. The Hilbert amplitude field used is mean-centered in panel A, and FP-centered in panel B. Panel C corresponds to quick estimates of amplitude fields and baseline noise level. Panel D corresponds to full estimates of amplitude fields and twice the baseline noise level. Panel C and D are based on a mean-centered Hilbert amplitude field. The color scale refers to differences in the average number of pulses delivered between stimulation based on isostable amplitude and on Hilbert amplitude. A positive difference (yellow to red) means that more pulses were delivered with the strategy based on isostable amplitude at this location. Results in each panels were obtained with three trials of 5000 s of stimulation.

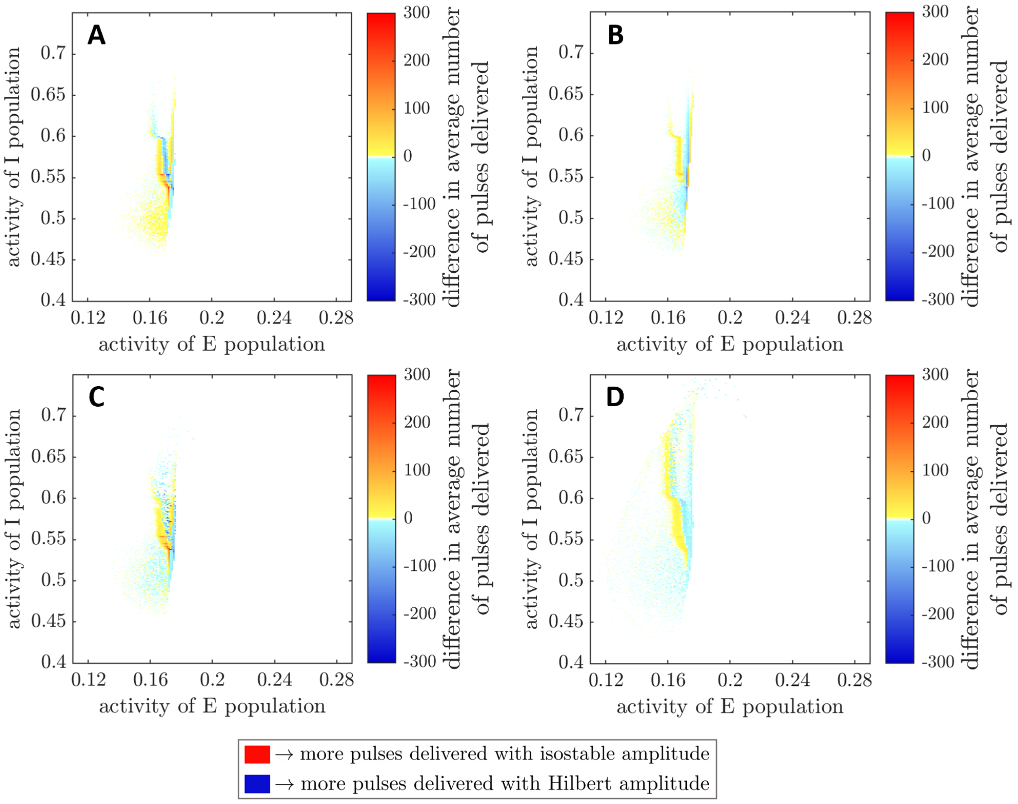

Figure J: Comparison of the average number of pulses delivered by phase space stimulation based on isostable amplitude and on Hilbert amplitude for patient 6, $b = 10$, $\delta E = 10\delta E_0$. Panel A and B correspond to full estimates of amplitude fields and baseline noise level. The Hilbert amplitude field used is mean-centered in panel A, and FP-centered in panel B. Panel C corresponds to quick estimates of amplitude fields and baseline noise level. Panel D corresponds to full estimates of amplitude fields and twice the baseline noise level. Panel C and D are based on a mean-centered Hilbert amplitude field. The color scale refers to differences in the average number of pulses delivered between stimulation based on isostable amplitude and on Hilbert amplitude. A positive difference (yellow to red) means that more pulses were delivered with the strategy based on isostable amplitude at this location. Results in each panels were obtained with three trials of 5000 s of stimulation.
